# Advanced Sensing System for Sleep Bruxism across Multiple Postures via EMG and Machine Learning

**DOI:** 10.3390/s24165426

**Published:** 2024-08-22

**Authors:** Jahan Zeb Gul, Noor Fatima, Zia Mohy Ud Din, Maryam Khan, Woo Young Kim, Muhammad Muqeet Rehman

**Affiliations:** 1 Department of Electronic Engineering, Maynooth University, W23A3HY Maynooth, Ireland; 2 Department of Biomedical Engineering, AIR University, Islamabad 44000, Pakistan; noorfatima.arshad26@gmail.com (N.F.); drzia@au.edu.pk (Z.M.U.D.); 3 Department of Electronic Engineering, Faculty of Applied Energy System, Jeju National University, Jeju 63243, Republic of Korea; maryamkhan93@stu.jejunu.ac.kr

**Keywords:** bruxism sensing, anatomical positions, machine learning, EMG acquisition, signal processing

## Abstract

Diagnosis of bruxism is challenging because not all contractions of the masticatory muscles can be classified as bruxism. Conventional methods for sleep bruxism detection vary in effectiveness. Some provide objective data through EMG, ECG, or EEG; others, such as dental implants, are less accessible for daily practice. These methods have targeted the masseter as the key muscle for bruxism detection. However, it is important to consider that the temporalis muscle is also active during bruxism among masticatory muscles. Moreover, studies have predominantly examined sleep bruxism in the supine position, but other anatomical positions are also associated with sleep. In this research, we have collected EMG data to detect the maximum voluntary contraction of the temporalis and masseter muscles in three primary anatomical positions associated with sleep, i.e., supine and left and right lateral recumbent positions. A total of 10 time domain features were extracted, and six machine learning classifiers were compared, with random forest outperforming others. The models achieved better accuracies in the detection of sleep bruxism with the temporalis muscle. An accuracy of 93.33% was specifically found for the left lateral recumbent position among the specified anatomical positions. These results indicate a promising direction of machine learning in clinical applications, facilitating enhanced diagnosis and management of sleep bruxism.

## 1. Introduction

Sleep bruxism (SB) is intricately linked to sleep rhythms, often disrupting the natural flow of sleep cycles. With 31.4% of the general population affected by sleep bruxism, this condition has emerged as a major health concern [1]. It is accompanied by a teeth-grinding sound, which involves rhythmic masticatory muscular activity (RMMA) occurring about once every second. Awake bruxism, which manifests as conscious clenching or grinding of the teeth during awake hours, has a prevalence of 22.1% [2]. In contrast, sleep bruxism, which happens at night, is often unbeknownst to the individual. The repetitive jaw muscle contractions linked with sleep bruxism can have dental and overall health consequences [3]. It might be a factor in dental problems such as temporomandibular joint pain, tooth degradation, and damage to dental restorations [4,5]. The severity, but not prevalence, of sleep bruxism was found to be higher in patients with hypertension, increased BMI, lower mean SpO2 values, and higher percentages of SpO2 below 90% [6].

Researchers are investigating a variety of techniques to address the specific challenges offered by this sleep-related disorder. Although there are many solutions available for Bruxism detection, no one provides a lightweight, easily integrated solution that can be incorporated with existing dental technology for daily usage. This paper contributes to the ongoing research by proposing a novel approach to bruxism detection that aims to be both efficient and user-friendly.

In this research, the effect of different anatomical positions associated with sleep is examined in the detection of sleep bruxism activity. EMG is used as a tool to record the voluntary contractions from the temporalis and masseter muscle in the supine and left and right lateral recumbent positions. The EMG data employed with machine learning classifiers facilitates the diagnosis of bruxism, which is a frequently underdiagnosed disease. This helps to mitigate the resulting consequences of the psychological and physical well-being of sleep bruxers.

This paper is organized as follows: Section 2 presents the related work, discussing previous research relevant to this study. Section 3 describes the methods used, detailing the experimental setup and procedures. Section 4 reports the results obtained from the research. Section 5 offers a discussion of the findings, interpreting the results in the context of the existing literature. Lastly, Section 6 concludes the paper, suggesting limitations and areas for future research.

## 2. Related Work

Several devices and techniques have been developed to detect and manage bruxism. Sidharth et al. [7] have developed e-Sense earbuds employing an inertial measurement unit IMU at the temporomandibular joint (TMJ). Similarly, Bondareva et al. [8] have designed an IMU-based device comprising an accelerometer and gyroscope. Pressure sensors are also utilized to measure the pressure generated within the ear canal [8,9,10]. An iBrux gadget has been developed by Emer O’Hare et al. [11] that is made up of a MEMS piezo-resistive pressure sensor (Bosch BMP280) and is placed at the buccal surface of the jaws (the outer surface of the teeth facing the cheeks). Bruxoff is a safe, simple screening device for evaluating masseter muscle activity during sleep, but its lack of a microphone or camera limits its diagnostic capabilities for sleep bruxism [12]. For detection of occlusal force signal, piezoresistive film is implanted in an oral device [13,14,15,16]. The design and assembly of the sensor integrated with dental splints is also very common for the treatment of bruxism [17,18,19,20]. While these devices have shown efficacy, it is important to consider the potential discomfort to patients during prolonged usage. Hence, there is a need for timely identification and diagnosis of bruxism without reliance on traditional devices. By identifying bruxism earlier and more affordably, we can intervene sooner, potentially mitigating the progression of the condition. Although such technology does not directly prevent bruxism from developing, it allows for timely intervention, which can prevent further dental damage or discomfort, effectively contributing to a broader management strategy.

Recent studies show that researchers are focusing more and more on employing machine learning (ML) classifiers for efficient detection of SB activity. Jirakittayakorn et al. [21] placed an EMG sensor on one ear pad to detect facial movements. Once the EMG data were collected, machine learning algorithms were applied to classify the bruxism activity [12]. A jaw clench was identified based on peak detection [18]. An ML-enabled device that uses CNN to remotely assess and diagnose sleep bruxism by classifying audio signals has also been designed [22]. Neural networks comprising autoregression and wavelet entropy have been designed for such a study [23]. A hybrid classifier [21] combines multiple machine-learning classifiers. The classification of sleep bruxism using the C4-P4 EEG channel were used [24] that achieved a maximum accuracy of 97% and a specificity of 90% [25], while another study reported an accuracy of 97.84% and specificity of 98.44% [26]. These advancements exemplify the promising potential of machine learning in enhancing the efficiency of sleep bruxism detection, leading to better diagnosis and treatment options.

The most practical and efficient technique for identifying masticatory muscle activity in bruxism is using electromyography (EMG) [27,28]. The primary masticatory muscles are the masseter, temporalis, and the medial and lateral pterygoid [29]. EMG-based detection systems for bruxism primarily focus on the contractions of the masseter muscle [10,19] due to its significant role in the jaw movements involved in teeth grinding and jaw clenching. The other prime masticatory muscle causing maximal contractions in teeth grinding is the temporalis muscle, which is not a center of focus in the context of detection-based systems for bruxism [24]. Hence, the reading taken from the masseter muscle alone may not give accurate results, as it overlooks the contributions of other masticatory muscles for bruxism assessment, particularly the temporalis.

Nonetheless, normal participants’ EMG data can be obtained to mimic the activity of teeth grinding and jaw clenching. Prior studies have shown that it is feasible to instruct subjects who do not have a diagnosis of sleep bruxism to clench and grind their teeth for data acquisition [6,10]. Using healthy controls helps establish baseline data and validate the experimental methodology before applying it to a clinical population. This approach ensures that the setup is reliable and effective. Moreover, this approach mitigates potential ethical concerns related to inducing stress or discomfort in sleep bruxers [30]. However, it is important to note that while healthy participants can simulate certain aspects of bruxism, they cannot replicate the full force and behavioral characteristics of bruxism, which often exceed voluntary capabilities. Recruiting individuals with sleep bruxism poses significant challenges, therefore making healthy subjects a more practical choice for initial feasibility studies.

Furthermore, research on sleep bruxism is constrained to muscle activity monitoring. The associated anatomical positions in individuals during sleep include supine, the left and right lateral recumbent positions, and the prone position. The focus of researchers in monitoring teeth grinding has been muscle activity [25,26] regardless of anatomical positions. The comparative analysis of primary muscles involved in bruxism (temporalis and masseter) during different anatomical positions associated with sleep has not yet been researched.

## 3. Materials and Methods

Electromyograms (EMGs) were acquired from subjects lying in the supine and left and right lateral recumbent positions using the BIOPAC MP36 Acquisition Unit [31], applying all the required digital filters. Once the signal of interest was acquired, it was further pre-processed before feature extraction and then fed into machine learning classifiers to detect bruxism. The remaining sections discuss the detailed methodology and results obtained.

### 3.1. Data Acquisition

The electromyography data were acquired from 10 healthy subjects between the ages of 18 to 25, with no prior history of bruxism. The subject selection process was intended to create gender balance, resulting in an equal number of male and female participants i.e., five. This gender parity improves the research’s capacity to generalize findings across varied demographic groups, resulting in a more comprehensive understanding of the research aims. 

**Figure 1:**
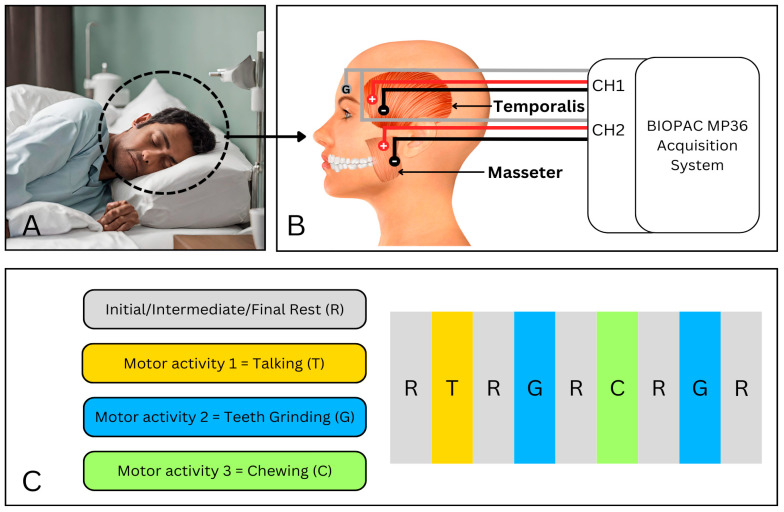
The overall methodology of this research. (**A**) shows a person doing jaw clenching during sleep. (**B**) shows the data acquisition using the EMG module from MP36 Biopac. The surface electrodes are placed on the two concerned muscles, i.e., the temporalis and masseter muscles. (**C**) The data were acquired from the patient lying in the supine and left and right lateral recumbent positions, following this paradigm with initial, intermediate, and final rest periods of 5 s each, and motor activities of 10 s. The motor activities performed in this experiment comprised all the possible motor movements associated with the two concerned muscles.

The study selection was performed to minimize bias while assuring the reliability and validity of the collected data following research ethics. The data were acquired repetitively across five trials for each anatomical position associated with sleep: the supine and left and right lateral recumbent positions, as shown in Figure 1. Informed consent was taken from all subjects before data collection. The study complied with ethical guidelines for medical research involving human subjects, as defined by the World Medical Association Declaration of Helsinki. 

**Figure 2:**
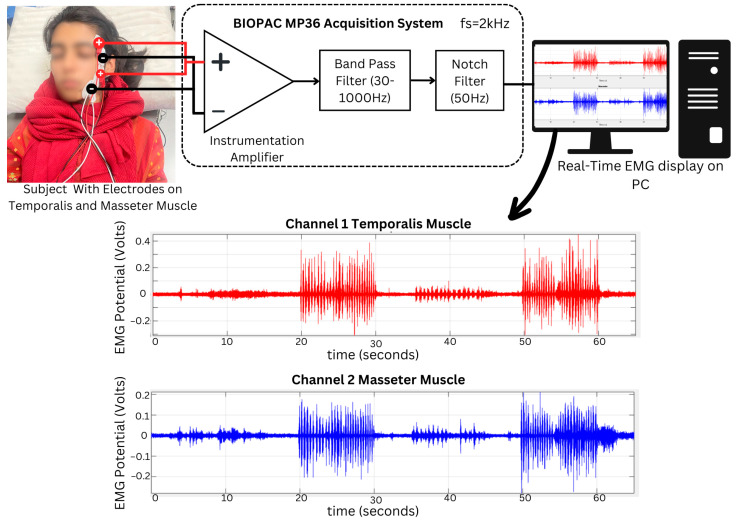
The EMG surface electrodes were placed on the temporalis and masseter muscles with common ground electrodes placed on the frontal bone. The temporalis muscle was set as channel 1 while the masseter muscle was set as channel 2. The signal was acquired using BIOPAC MP36, which mainly comprises an instrumentation amplifier to capture the signal with a high pass filter of 30 Hz and a low pass filter of 1000 Hz. The data acquired were displayed on the PC in real-time from two channels, which were further stored to perform signal processing and feature extraction.

The experimental setup used for EMG data acquisition is shown in Figure 2. The MP36 Four-Channel Data Acquisition System of Biopac Student Lab Basic System [31] was used to acquire the desired EMG signal. An inbuilt microprocessor-controlled data acquisition and communication with the computer in the MP36 unit. Among four channels, two channels were simultaneously used to acquire EMG signals from two desired muscles. The sampling frequency of both channels was set to be 2 kHz (2000 samples per second). In addition to BIOPAC fixed hardware filters, digital IIR filters with desired meaningful frequencies were selected in Biopac Student Lab 3.7.3 software. This was necessary to minimize the noise in the acquired EMG signal. Hence, the signals obtained from the muscles were within the range of 30–1000 Hz for high-pass and low-pass Butterworth filters. The notch frequency was set at 50 Hz to filter the possible power line noise. The BIOPAC MP36 data acquisition unit employs a switchable bank of single pole high pass filters for each channel which are generally first-order filters. Once the preliminary acquisition system settings were finalized, pre-gelled surface electrodes of Ag/AgCl were used to collect EMG signals derived from the temporalis (channel 1), labeled as red in Figure 2, and the masseter (channel 2) muscles, labeled as blue in Figure 2, simultaneously. 

**Figure 3:**
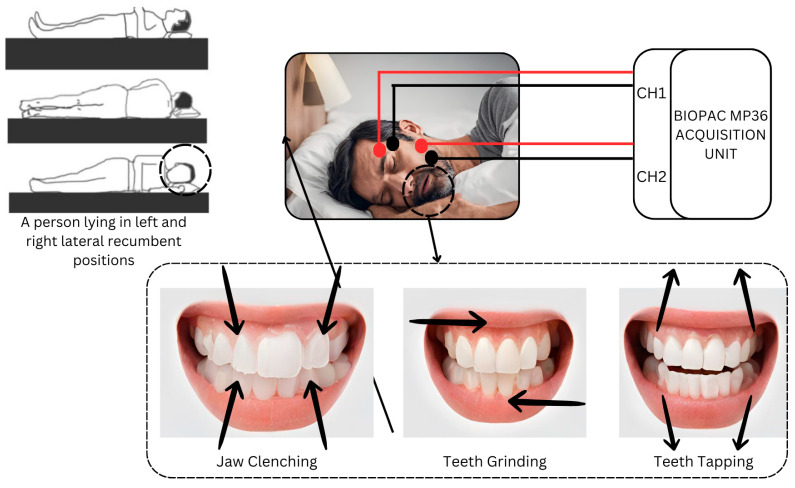
The subjects lay first in the supine position and then in the left and right lateral recumbent positions and performed bruxism activities associated with teeth grinding, jaw clenching, and teeth tapping as indicated with arrows while EMG electrodes were placed on the temporalis and masseter muscles on the right side of the patient.

The unilateral approach of placing electrodes on either the dominant or non-dominant side (right or left) may hinder a comprehensive understanding of muscle coordination and symmetry. Hence, the similarity in muscle contraction patterns was studied beforehand by acquiring EMG bilaterally, from both sides. This was necessary to avoid any potential biases in the result and to provide a generalization of this research. Once the data were acquired bilaterally, correlation was performed to check the similarity of muscle contraction patterns of the temporalis muscle of the left and right sides and the masseter muscle of the left and right sides to avoid any ambiguity. This approach aims to identify if any of the left side or right side is more dominant or to identify any significant differences in muscle activation patterns. Previous studies have shown that bilateral EMG recording can provide valuable insights into muscle coordination and symmetry, supporting the approach in our study [32]. The subject’s skin was wiped with alcohol to ensure the anti-septicity of the skin surface. Then, the subjects were instructed to lie in a supine position first, then the left lateral recumbent position, and lastly, the right lateral recumbent position, as shown in Figure 3. All subjects were instructed to experiment in the specified order. The experimental paradigm was designed using PsychoPy 2023.2.3 software. This was used to guide subjects about the procedure of data acquisition with graphical illustrations of the motor activities involved, and for precise time control between rest and motor activities in our experiment. 

The BIOPAC acquisition unit recorded the EMG data continually for 65 s with separate files for each anatomical position, so the obtained signal had segments of motor activity for the temporalis and masseter muscle along with no-action (resting) segments in the supine and left and right lateral recumbent positions. As a result, 150 data files were obtained for each of the two muscles in the experiment, comprising segments of rest, talking, rhythmical chewing, and jaw clenching/teeth grinding. With each subject performing the experiment across 5 trials (i.e., 10 subjects × 5 trials = 50) and considering three different positions ((a) supine, (b) left lateral recumbent, and (c) right lateral recumbent), we obtained 50 × 3 = 150 data files (i.e., 50 files for each of three positions), with each file containing 130,000 samples.

### 3.2. Feature Extraction

After the raw data were acquired, further processing and analysis was performed using MATLAB R2023a software. 

**Figure 4:**
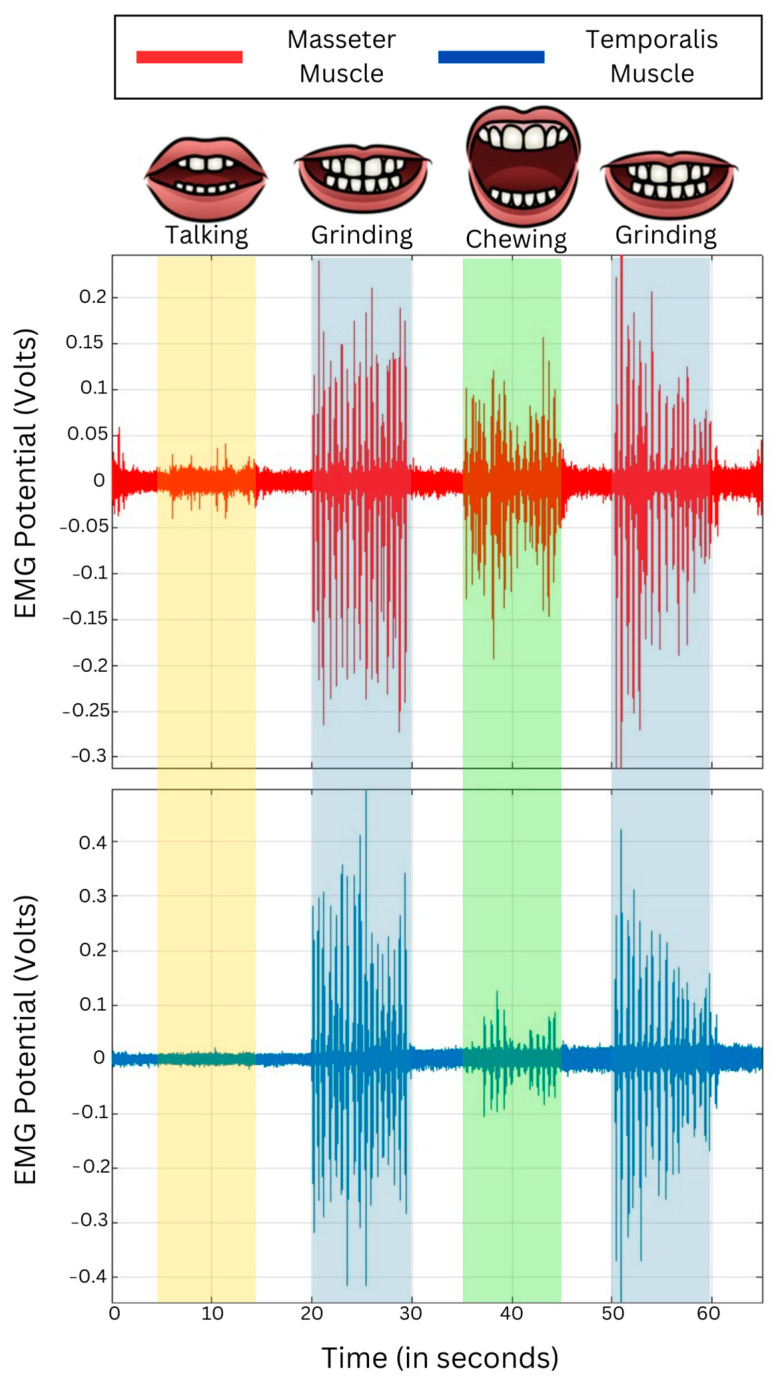
The MATLAB plot of EMG acquired in 65 s from the temporalis muscle and masseter muscles demonstrates the segmented blocks based on the sampling rate of 2000 Hz. Each rest block i.e., initial, intermediate, and final, is 5 s in length, and each motor activity block is 10 s in length.

**Figure 5:**
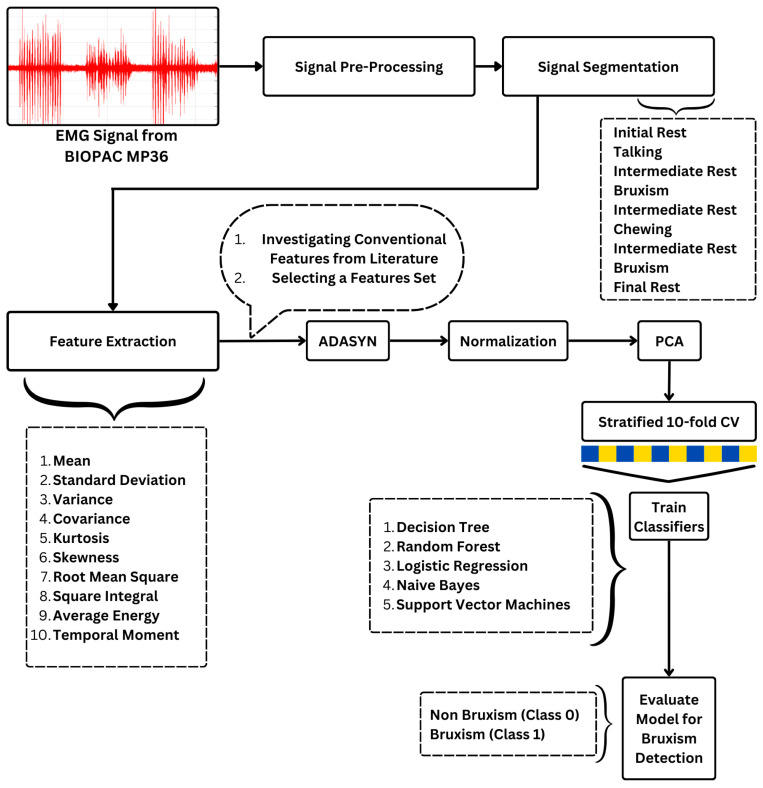
The signal in MATLAB was subjected to pre-processing before feature selection and feature extraction. The features were selected based on extensive literature research related to our application. A total of 10 features were selected, which were then fed as input to the machine learning classifiers. The Adaptive Synthetic Sampling Approach was applied to handle the imbalance of binary classes. The features were then reduced using principal component analysis and stratified 10-fold cross-validation was applied to divide the dataset into split, test, and valid sets. The models were then trained, and their performance was evaluated for bruxism detection.

MATLAB is preferred for its comprehensive graphical interface and specialized toolboxes for signal processing, making it straightforward to preprocess EMG data. A digital IIR 10th-order Butterworth band pass filter at 150–300 Hz was applied to the signal before feature extraction. This was performed to ensure the removal of noise present in the EMG signal, since it affects the efficiency of the classifier. After pre-processing, the EMG signal was segmented into different blocks based on the sampling rate. A visual illustration is shown in Figure 4. The resting segments, i.e., (a) initial rest, (b) intermediate rest between each motor activity, and (c) final rest, each had 5 s × 2000 = 10,000 samples. Each segment of motor activity of the temporalis and masseter muscles, involving (a) talking, (b) chewing, and (c) bruxism, had 10 s × 2000 = 20,000 samples. Once the pre-processing and segmentation had been completed, the EMG Feature Extraction Toolbox was used [33] to extract the features as named in Figure 5. The integrated toolboxes in MATLAB allow for a smooth transition from signal processing to feature extraction. Specifically, after segmenting the EMG signals, a set of time-domain functions was applied to each segment to extract relevant features, which were then used as inputs for the machine learning classifiers. A total of 10 time domain features were selected for feature extraction [33]. Time domain features were utilized because of their simple calculation and the fact that they do not require any transformation, providing an effective representation of the EMG signal. These features are based on signal amplitude and are useful in pattern recognition and detection systems based on electromyography (EMG).

### 3.3. Machine Learning Classifiers

Once the features were extracted, the labeled data with 10 input features and 2 o/p classes was fed into the following machine learning algorithm shown in the Figure 5. The machine learning classifiers employed in this study were logistic regression, naïve Bayes, decision tree, random forest, and support vector machine. The total number of samples fed into the classifier was 3250 from 150 data records of 10 subjects across five trials in the supine and left and right lateral recumbent positions, as described in Section 3.1. Since the output label count resulted in imbalance of classes, with 69% being labeled as non-bruxism and 31% being labeled as bruxism, the ADASYN (Adaptive Synthetic Sampling Approach) was used to address this issue. ADASYN was applied only to the training set to avoid data leakage and ensure that synthetic samples did not affect the validation or test sets. Following the oversampling, features were scaled using Standard Scaler. Principal Component Analysis (PCA) was then performed to reduce dimensionality while retaining 95% of the variance. To evaluate the model, Stratified K-Fold Cross-Validation (10 folds) was implemented, ensuring that each fold maintained the overall class distribution. The classifiers’ accuracies in terms of detecting bruxism were then obtained. This approach provided a reliable performance estimate while balancing the risks of overfitting and underfitting.

## 4. Results

This section describes the comparative analysis of electromyograms and classification results obtained from five machine-learning classifiers for bruxism detection based on EMG data collected in the left and right lateral recumbent positions.

### 4.1. Correlation of Bilateral Masticatory Muscles 

The placement of electrodes for electromyography (EMG) on muscles on the either right or left side of the body can affect the generalization of results over large groups and the comprehensiveness of the research conducted. While the human body is generally symmetrical, there can be slight anatomical differences between the right and left sides. Considering this fact, EMG electrodes were placed on the specified masticatory muscles, the temporalis and the masseter, and their comparison was studied against bilateral symmetry. The extracted time domain features were compared for the temporalis and masseter muscles of the left and right sides, as shown in Table 1.

These results indicate that the accuracy was consistent and reliable regardless of whether the left or right muscle was used for the detection of sleep bruxism, with random forest and support vector machines outperforming other classifiers used. To further validate the reliability and similarity of extracted features of EMG of left- and right-side masticatory muscles, similarity scores were obtained by performing basic statistical analysis using Python. The similarity scores were used to compare the activity patterns of the two muscles based on the extracted features of mean, standard deviation, variance, co-variance, kurtosis, skewness, root mean square, square integral, average energy, and temporal moment. The mean correlation values between the left and right temporalis and masseter muscles, considering the same anatomical position, are tabulated in Table 2.

**Figure 6:**
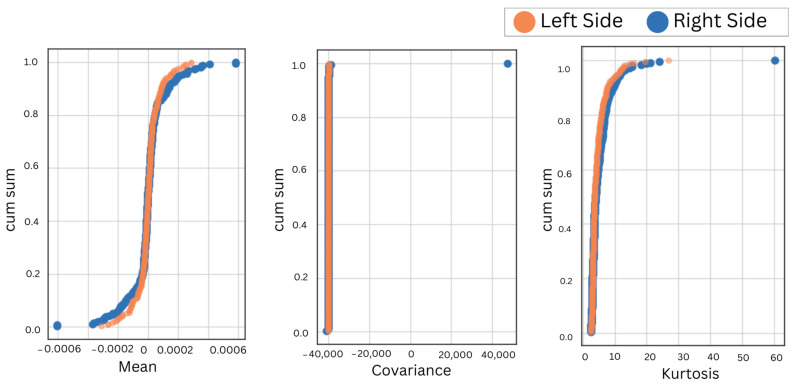
The CumSum plots for bilateral comparison of the temporalis muscle in the left lateral recumbent position.

**Figure 7:**
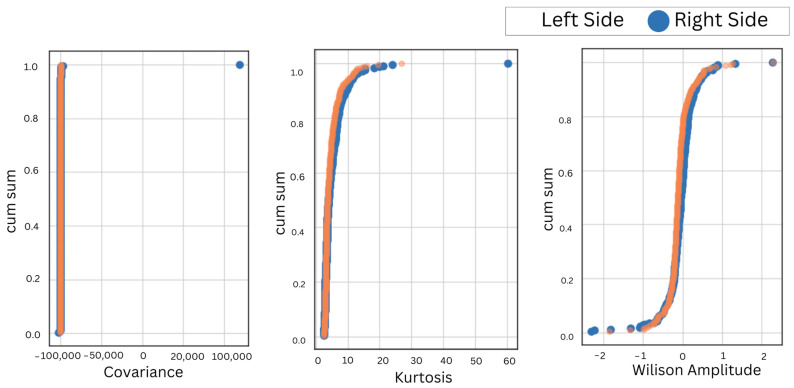
The CumSum plots for bilateral comparison of the temporalis muscle in the right lateral recumbent position.

**Figure 8:**
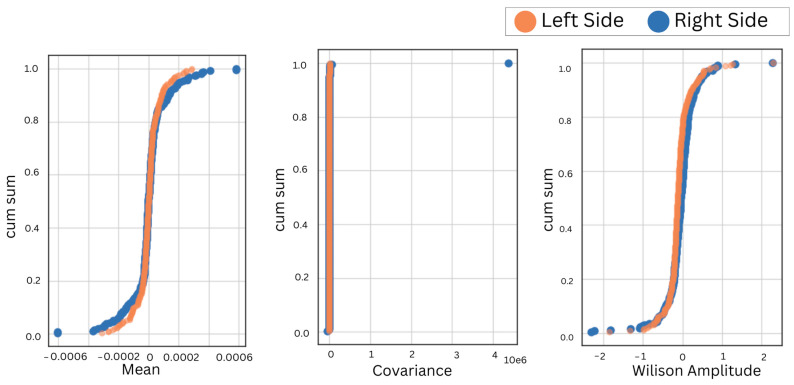
The CumSum plots for the bilateral comparison of the masseter muscle in the left lateral recumbent position.

**Figure 9:**
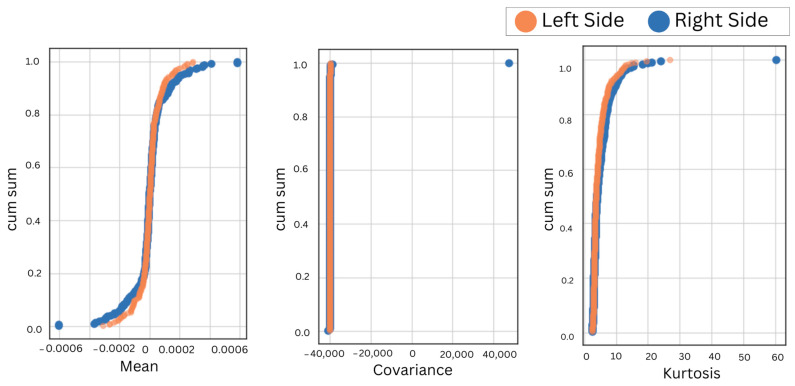
The CumSum plots for bilateral comparison of the masseter muscle in the right lateral recumbent position.

A positive correlation between bilateral muscles irrespective of the anatomical position validates the similarity of EMG features in bruxism detection, also irrespective of the placement of electrodes. Furthermore, the cumulative sum over reduced features of the left and right muscles in the left and right lateral recumbent positions was obtained to visualize the overall discrepancy in the performance and behavior of bilateral muscles, as shown in Figure 6, Figure 7, Figure 8 and Figure 9.

### 4.2. Frequency Analysis

**Figure 10:**
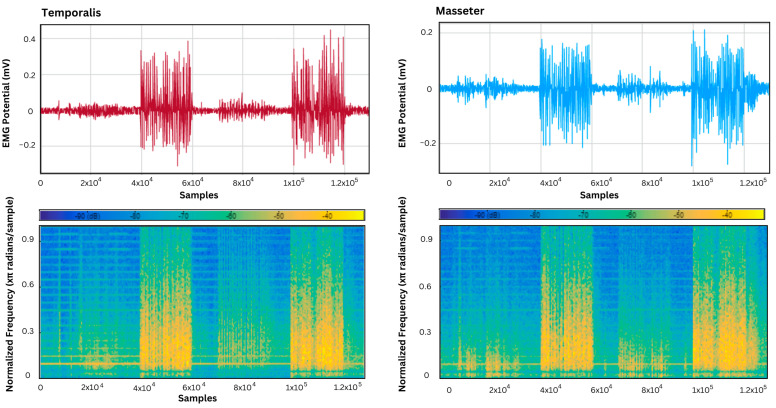
The power spectrum plot for the emporalis muscle in the supine position, and masseter muscle in the supine position.

**Figure 11:**
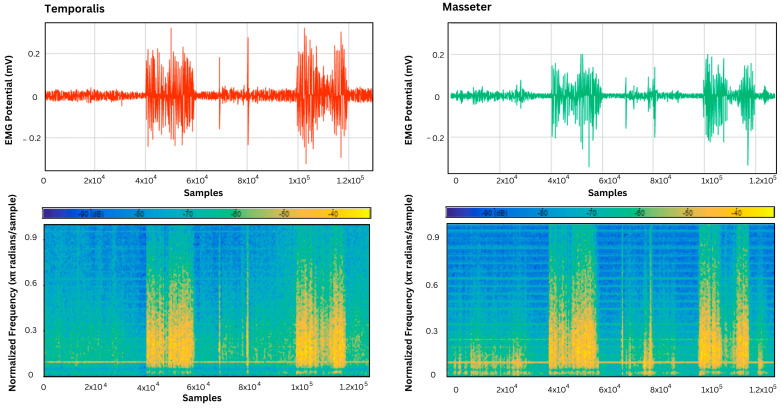
The power spectrum plot for the temporalis muscle in the left lateral recumbent position, and the masseter muscle in the left lateral recumbent position.

**Figure 12:**
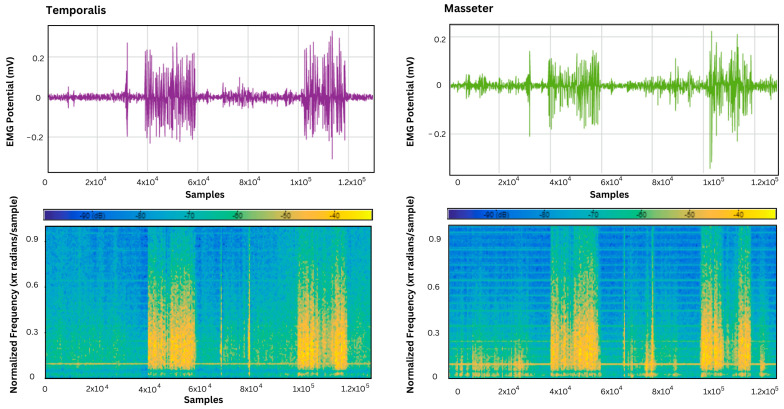
The power spectrum plot for the temporalis muscle in the right lateral recumbent position, and the masseter muscle in the left lateral recumbent position.

The acquired EMG signals from the temporalis and masseter muscles were analyzed using a signal analyzer app on MATLAB to assess the behavior of specified muscle activity over different frequencies. This was conducted to identify the dominant frequency components that correspond to the rate of muscle activation, which was at its maximum in the samples associated with teeth grinding and jaw clenching, as shown in Figure 10, Figure 11 and Figure 12.

### 4.3. Evaluation Metrics

The evaluation parameters to evaluate model performance were selected to be accuracy, recall, precision, and F1-score. The values of evaluation metrics for decision tree (DT), random forest (RF), logistic regression (LR), naïve Bayes (NB), and support vector machine (SVM) were obtained for the muscles under study.

#### 4.3.1. Evaluation Metrics for Temporalis Muscle

The evaluation parameters for the temporalis muscle in the supine, left lateral recumbent, and right lateral recumbent positions are tabulated in Table 3.

#### 4.3.2. Evaluation Metrics for Masseter Muscle

The evaluation parameters for the masseter muscle in the supine, left lateral recumbent, and right lateral recumbent positions are tabulated in Table 4.

### 4.4. Comparison of Accuracies of Temporalis and Masseter Muscle

The comparison between the temporalis and masseter muscles in terms of finer detection of sleep bruxism is shown in Figure 6.

### 4.5. Confusion Matrix

The confusion matrices for the SVM model for the temporalis and masseter muscles are shown in Figure 7. The matrix made it possible to compare each class by identifying and quantifying instances of classifications focusing on true positives and true negatives. 

## 5. Discussion

During motor activities, the right and left sides of the body may exhibit different movement patterns or compensations, leading to variations in muscle activation and EMG readings. It is important to consider the bilateral masticatory muscles for bruxism detection. It ensures that conclusions drawn from the research apply to a diverse group of people. Hence, the EMG electrodes were placed on the bilateral sides of the temporalis and masseter muscles in the left and right lateral recumbent positions. First, the accuracies for different selected machine learning classifiers were obtained, as shown in Table 1. This indicated a consistent result despite the placement of electrodes on either the left- or right-side muscles in different anatomical positions. Next, to validate further the correlation of bilateral sides in both anatomical positions were obtained for the temporalis and masseter muscles, as shown in Table 2. Hence, the correlation of the EMG for the temporalis and masseter muscles was obtained, as shown in Table 2. The cumulative sum of reduced features was plotted to visualize the trend in similarity in activation patterns of the temporalis and masseter muscle on the left and right sides. Since the results showed maximum correlation, the EMG data from further subjects were acquired from either side to compare the efficacy of the two primary masticatory muscles associated with bruxism in three different anatomical positions related to sleep. This approach was supported by the consistent correlation observed, but the initial bilateral placement remains crucial for ensuring comprehensive data collection and analysis.

Furthermore, the EMG signals in the supine, left lateral recumbent, and right lateral recumbent positions were analyzed using the Signal Analyzer App on MATLAB. This was performed to identify the dominant frequencies associated with muscle contractions. As shown in Figure 10, Figure 11 and Figure 12, among the masticatory muscle activities performed (talking, chewing, and teeth grinding), the maximum power was exhibited during the activities of bruxism, as marked by the presence of yellow in the power spectrum while talking, and rest activities showed the least power associated with muscle activation patterns. This proves the efficacy of the temporalis muscle in differentiating the performed activities of chewing and talking from teeth grinding, while the masseter muscle was also active in the activity of talking, unlike the temporalis muscle. 

**Figure 13:**
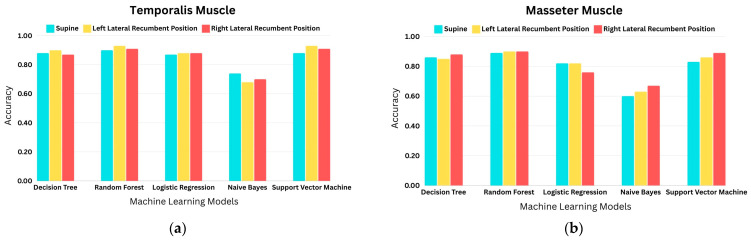
Comparison of accuracies of machine learning models at supine left lateral recumbent and right lateral recumbent positions for the masticatory muscles: (**a**) temporalis; (**b**) masseter.

The evaluation metrics of the temporalis and masseter muscle in the supine, left lateral recumbent, and right lateral recumbent positions for the selected classifiers are tabulated in Table 3 and Table 4. The evaluation metrics provide accuracy, recall, precision, and f1-score for all classifiers. In this case, the preferred choice of evaluation metric parameter is accuracy. As the bruxism and non-bruxism classes were balanced after applying SMOTE, accuracy, which represents the correctness of predictions, can be the best statistic for truly balanced classes. This is because it equally considers the correct predictions for both classes, without bias towards any particular class. In this research, the temporalis muscle gave recall of 0.90, 0.93, and 0.91 in the supine, left lateral recumbent, and right lateral recumbent positions, respectively, for random forest. Similarly, the masseter muscle gave recall of 0.89, 0.90, and 0.90 in the supine, left lateral recumbent, and right lateral recumbent positions, respectively, for random forest. This demonstrated the efficiency of random forest in the detection of sleep bruxism as compared to other classifiers. Better accuracies were achieved using the temporalis muscle, as shown in Figure 13. While bruxism is associated with the motor activity of masticatory muscles, which are the temporalis, masseter, and lateral and medial pterygoid, the prime masticatory muscles involved in the movements of the jaw are the temporalis and masseter. For jaw clenching, the comparative analysis of the temporalis and masseter muscles yielded that the temporalis muscle provides better accuracy in the detection of sleep bruxism, which has not yet been explored by researchers. 

**Figure 14:**
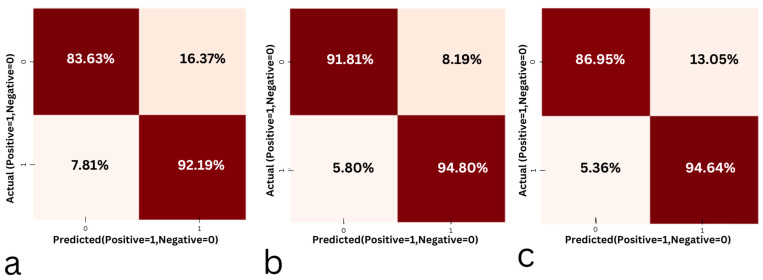
The confusion matrices from the support vector machine model for (**a**) the temporalis muscle in a supine position, (**b**) the temporalis muscle in a left lateral recumbent position, and (**c**) the temporalis muscle in a right lateral recumbent position.

As the temporalis muscle outperformed the masseter muscle in bruxism detection when a random forest classifier was employed, the confusion matrix of random forest for bruxism detection in the supine and left and right lateral recumbent positions was obtained, as shown in Figure 14. This was obtained in order to assess the overall performance of the random forest classifier in truly predicting bruxism in sleep bruxers. The confusion matrix consisted of the true positives (TP), or the correct classification of teeth grinding/jaw clenching as bruxism; the true negatives (TN), or the correct classification of talking/chewing/resting as non-bruxism; false positives (FP), or the incorrect classification of non-bruxism activities as bruxism; and false negatives (FN), or the incorrect classification of teeth grinding and jaw clenching as non-bruxism. The obtained confusion matrices evaluated the efficiency of random forest in bruxism detection with high TP and FN.

These results reveal that sleep position has a significant impact on bruxism recognition, as the detection accuracy varied among the supine and the left and right lateral recumbent positions, with the left lateral recumbent position exhibiting the highest accuracy in the detection of the orofacial responses of bruxism. Additionally, among the two masticatory (temporalis and masseter) muscles involved, the temporalis muscle consistently provided more accurate and reliable signals for bruxism detection. 

## 6. Conclusions

In this research, a multimodal set of machine learning algorithms, including decision tree, random forest, logistic regression, naïve Bayes, and support vector machine, were employed for sleep bruxism detection. The machine learning models were trained on extracted features of EMGs acquired from two prime mastication muscles, i.e., the temporalis and masseter, in the supine and left and right lateral recumbent positions. The random forest algorithm showed relatively better performance in predicting orofacial behavior associated with sleep bruxism. Our findings indicate that sleep position affects the recognition of bruxism. Specifically, the detection accuracy varied across the supine and left and right lateral recumbent positions, with the left lateral recumbent position showing the highest accuracy. Moreover, when comparing the EMG data from the temporalis and masseter muscles, the temporalis muscle consistently provided more accurate and reliable signals for bruxism detection. To put it briefly, we achieved the highest accuracy with the temporalis muscle in the left lateral recumbent position, at 93.3%. However, this study had certain limitations. It was conducted in a controlled environment with healthy participants rather than individuals diagnosed with sleep bruxism. Future research should address these limitations by including a clinical population and expanding the sample size. Additionally, exploring other sleep positions could help generalize the findings to broader populations.

## Figures and Tables

**Table 1:** Accuracies of temporalis and masseter muscles in left lateral and right lateral recumbent positions with electrodes placed on bilateral muscles.

Anatomical Position	Machine Learning Model	Temporalis		Masseter	
		Left	Right	Left	Right
Left Lateral Recumbent Position	Decision Tree	0.8472	0.8056	0.75	0.7917
	Random Forest	0.8611	0.8611	0.8194	0.8333
	Naïve Bayes	0.75	0.7917	0.7222	0.75
	Logistic Regression	0.8194	0.8472	0.8333	0.8194
	Support Vector Machines	0.8333	0.8611	0.8333	0.8333
Right Lateral Recumbent Position	Decision Tree	0.875	0.8472	0.8472	0.8333
	Random Forest	0.8889	0.9028	0.875	0.9167
	Naïve Bayes	0.875	0.8889	0.8333	0.875
	Logistic Regression	0.9028	0.9167	0.8194	0.9167
	Support Vector Machines	0.8889	0.9167	0.8472	0.9028

**Table 3:** Accuracy, precision, recall, and f1-score for the temporalis muscle in the supine, left lateral recumbent, and right lateral recumbent positions.

Anatomical Position	Machine Learning Model	Accuracy	Precision	Recall	F1-Score
Supine Position	Decision Tree	0.84	0.85	0.83	0.84
	Random Forest	0.87	0.84	0.94	0.88
	Logistic Regression	0.80	0.84	0.75	0.79
	Naïve Bayes	0.76	0.84	0.66	0.74
	Support Vector Machines	0.84	0.83	0.87	0.85
Left Lateral Recumbent Position	Decision Tree	0.89	0.88	0.89	0.89
	Random Forest	0.93	0.90	0.96	0.93
	Logistic Regression	0.87	0.89	0.86	0.87
	Naïve Bayes	0.65	0.83	0.39	0.53
	Support Vector Machines	0.92	0.90	0.94	0.92
Right Lateral Recumbent Position	Decision Tree	0.85	0.83	0.87	0.85
	Random Forest	0.89	0.86	0.93	0.89
	Logistic Regression	0.84	0.84	0.83	0.84
	Naïve Bayes	0.72	0.83	0.53	0.65
	Support Vector Machines	0.89	0.86	0.91	0.88

**Table 2:** Correlation of temporalis and masseter muscles in left lateral and right lateral recumbent positions with electrodes placed on bilateral muscles.

Anatomical Position	Left and Right Temporalis	Left and Right Masseter
Left Lateral Recumbent Position	0.9378	0.9555
Right Lateral Recumbent Position	0.9583	0.9262

**Table 4:** Accuracy, precision, recall, and f1-score of masseter muscle at supine, left lateral recumbent, and right lateral recumbent positions.

Anatomical Position	Machine Learning Model	Accuracy	Precision	Recall	F1-Score
Supine Position	Decision Tree	0.83	0.81	0.87	0.84
	Random Forest	0.86	0.83	0.92	0.87
	Logistic Regression	0.76	0.76	0.77	0.77
	Naïve Bayes	0.62	0.70	0.47	0.56
	Support Vector Machines	0.77	0.75	0.83	0.79
Left Lateral Recumbent Position	Decision Tree	0.83	0.84	0.83	0.84
	Random Forest	0.88	0.86	0.92	0.89
	Logistic Regression	0.65	0.85	0.69	0.77
	Naïve Bayes	0.78	0.87	0.37	0.52
	Support Vector Machines	0.86	0.85	0.89	0.87
Right Lateral Recumbent Position	Decision Tree	0.85	0.84	0.85	0.85
	Random Forest	0.90	0.85	0.96	0.90
	Logistic Regression	0.76	0.77	0.71	0.74
	Naïve Bayes	0.48	0.48	0.99	0.64
	Support Vector Machines	0.88	0.84	0.94	0.89

## Data Availability

Data will be available on reasonable request from the corresponding author.
